# Prevalence and duration of prescribed opioid use during pregnancy: a cohort study from the Quebec Pregnancy Cohort

**DOI:** 10.1186/s12884-021-04270-x

**Published:** 2021-12-01

**Authors:** Jin-Ping Zhao, Christelle Berthod, Odile Sheehy, Behrouz Kassaï, Jessica Gorgui, Anick Bérard

**Affiliations:** 1 Research Center, CHU Sainte-Justine, 3175, Chemin de la Côte-Sainte-Catherine, Montreal, Quebec H3T 1C5 Canada; 2 Faculty of Pharmacy, University of Montreal, Montreal, Quebec Canada; 3 EPICIME-CIC 1407 Lyon, Inserm, Pharmacotoxicology Department, CHU-Lyon, 69677 Bron, France; 4 University of Lyon 1, 69008 Lyon, France; 5 Laboratoire de Biométrie et Biologie Evolutive, University of Lyon 1; CNRS, UMR 5558, 69622 Villeurbanne, France

**Keywords:** Prescribed opioids use during pregnancy, Weak opioid codeine, Strong opioid morphine, hydromorphone, and oxycodone, Duration of opioid treatment

## Abstract

**Background:**

Recent studies show a rapid growth among pregnant women using high potency opioids for common pain management during their pregnancy. No study has examined the duration of treatment among strong opioid users and weak opioid users during pregnancy. We aimed to investigate the prevalence of prescribed opioid use during pregnancy, in Quebec; and to compare the duration of opioid treatment between strong opioid users and weak opioid users.

**Methods:**

Using the Quebec Pregnancy Cohort (1998–2015), we included all pregnancies covered by the Quebec Public Prescription Drug Insurance Program. Opioid exposure was defined as filled at least one prescription for any opioid during pregnancy or before pregnancy but with a duration that overlapped the beginning of pregnancy. Prevalence of opioids use was calculated for all pregnancies, according to pregnancy outcome, trimester of exposure, and individual opioids. The duration of opioid use during pregnancy was analyzed according to 8 categories based on cumulative duration (< 90 days vs. ≥90 days), duration of action (short-acting vs. long-acting) and strength of the opioid (weak vs. strong).

**Results:**

Of 442,079 eligible pregnancies, 20,921 (4.7%) were exposed to opioids. Among pregnancies ending with deliveries (*n* = 249,234), 5.4% were exposed to opioids; the prevalence increased by 40.3% from 3.9% in 1998 to 5.5% in 2015, more specifically a significant increase in the second and third trimesters of pregnancy. Weak opioid, codeine was the most commonly dispensed opioid (70% of all dispensed opioids), followed by strong opioid, hydromorphone (11%), morphine (10%), and oxycodone (5%). The prevalence of codeine use decreased by 47% from 4.3% in 2005 to 2.3% in 2015, accompanied by an increased use of strong opioid, morphine (0.029 to 1.41%), hydromorphone (0.115 to 1.08%) and oxycodone (0.022 to 0.44%), from 1998 to 2015. The average durations of opioid exposure were significantly longer among pregnancies exposed to strong opioid as compared to weak opioid regardless of the cumulative duration or duration of action (*P* < 0.05).

**Conclusions:**

Given the differences in the safety profile between strong opioids and the major weak opioid codeine, the increased use of strong opioids during pregnancy with longer treatment duration raises public health concerns.

**Supplementary Information:**

The online version contains supplementary material available at 10.1186/s12884-021-04270-x.

## Background

Pain during pregnancy is common as low back pain and pelvic pain may occur in over 70% of pregnancies [1, 2]. Opioids reduce the intensity of pain signal perception and over the past 20 years, the prevalence of prescribed opioids use during pregnancy has increased considerably [3–13].

Based on recent US national studies, 12.9% of privately insured pregnant women [3] and 22.8% of Medicaid insured pregnant women [4] filled at least one prescription for an opioid during their pregnancy. Given the increase in maternal age and associated co-morbidities and chronic pain, the use of opioids in pregnancy is expected to increase consequently [14, 15].

With the rise in the prevalence of opioid use during pregnancy, we can also note a sharp increase in the rate of infants with neonatal abstinence syndrome [16–18]. Of note, two in three infants with neonatal abstinence syndrome was exposed to prescribed opioids during gestation [17, 19]. Associations between maternal use of opioids in pregnancy and increased risk for birth defects [20–24] such as congenital heart defects [20, 21], spina bifida [10, 20], and oral clefts [24] have been reported in recent studies.

In Canada, the patterns of use of opioids during pregnancy varies between provinces. In Manitoba, the proportion of women who used prescribed opioids during their pregnancy went from 7.3% in 2001 to 7.7% in 2013 [8]. Overtime, there was a 4.3-fold increase in the mean morphine equivalents (MEQ) per pregnancy (284 mg in 2001 and 1218 mg in 2013) [8], as women had used higher potency opioids during their pregnancy. Bateman et al. [3] also reported a 31.3% increase in the prevalence of strong opioid oxycodone use, from 1.6% in 2005 to 2.1% in 2011; while a 28.4% decrease in the prevalence of weak opioid codeine use, from 7.2% in 2005 to 5.3% in 2011, among privately insured US pregnant women during their pregnancy. However, high potency opioids are not recommended for chronic noncancer pain given the lack of evidence concerning their effectiveness in other contexts, and tend to be associated with higher rates of overdose, misuse, addiction, and side effects [25–28].

In Quebec, the prevalence of the use of highly potent opioids, such as hydromorphone and morphine have been increasing from 2006 to 2016, while the use of low potent opioids such as codeine decreased [29]. It is unknown if these trends of opioid use in Quebec’s general population can be observed among pregnant women specifically. To date, no study has examined the duration of treatment among strong opioid users compare to weak opioid users during pregnancy. Therefore, we aimed to determine the prevalence of prescribed opioid use during pregnancy, in Quebec, and to compare the duration of opioid treatment between strong opioid users and weak opioid users.

## Methods

### Setting and study design

We analyzed data from the Quebec Pregnancy Cohort (QPC), which is an ongoing population-based cohort with prospective data collection on all pregnancies covered by the Quebec Public Prescription Drug Insurance, from 01/01/1998 to 12/31/2015, in the province of Quebec, Canada [30, 31]. The QPC is updated regularly; the latest data available at present are until 2015. Information for each pregnancy was obtained from province-wide databases and linked using unique personal identifiers. The QPC was constructed by identifying all pregnancies in the Régie de l’Assurance Maladie du Québec (RAMQ) and the provincial hospitalization archives database (MedEcho). The first day of the last menstrual period (LMP, defined as the first day of gestation) was determined using data on gestational age, which has been validated against ultrasound measures from patients’ charts [32]. Prospective follow-up data were available from 1 year before LMP, during pregnancy and until 12/31/2015.

The QPC data sources include the medical claims database (RAMQ: outpatient diagnoses, medical procedures, socio-economic status), Quebec Public Prescription Drug Insurance database (drug name, formulation, dosage, quantity dispensed, date of the dispensation, duration of the treatment, and days supplied), hospitalization archive database (MedEcho: in-hospital diagnoses and procedures, gestational age), and Quebec Statistics database (ISQ: patient socio-demographics, gestational age, birth weight). QPC data on prescriptions filled [33], gestational age [32] and birth weight [32] have been validated. The RAMQ medication database in the QPC represents 36% of women between 15 and 45 years of age in the province of Quebec, Canada [34]. Validation studies have shown that publicly insured pregnant women have similar characteristics and co-morbidities with those who have private medication insurance [35].

This study was approved by the CHU Sainte-Justine Institutional Review Board. The Quebec “Commission d’Accés à l’information” authorized database linkages.

### Participants

We included all pregnancies continuously covered by the Quebec Public Prescription Drug Insurance for ≥1 year before LMP, and during pregnancy.

### Opioid exposure

We identified opioid prescriptions (Table S1 A) filled from the Quebec Public Prescription Drug Insurance database, using timing of exposure determined by the dispensed date and duration of treatment (days supplied). Pregnancies were considered as opioid exposed if women had filled at least one prescription for any type of opioid during pregnancy or if they had filled a prescription before pregnancy but with a duration that overlapped the beginning of pregnancy. Individual opioids considered in our analyses included morphine, hydromorphone, oxycodone, fentanyl, codeine, pentazocine, meperidine, tapentadol, propoxyphene, tramadol and opioid formulas mixed with acetaminophen. The two opioid substitutes available in Quebec, methadone and buprenorphine-naloxone were also included in the estimation of prevalences. Since opioid substitutes were considered to have a different usage pattern with opioid analgesics, they were excluded for the analyses of indications and predictors of opioid exposure during pregnancy.

The overall prevalence of opioid use during pregnancy was calculated by dividing the number of pregnancies receiving at least one opioid during pregnancy by the total number of pregnancies in the cohort. The end of pregnancy was defined as the calendar date of a planned abortion, miscarriage, or delivery. The prevalence of opioid use in each trimester was calculated among pregnancies ending with a delivery. First trimester was defined as 0–14 completed weeks of gestation, second trimester 15–25 completed weeks of gestation, and third trimester from 26 weeks until delivery. When the duration of an opioid prescription overlapped trimesters, pregnancies were defined as exposed in both trimesters.

The opioids were categorized as strong or weak based on the Canadian Guideline for Safe and Effective use of Opioids [25, 26]. Strong opioids included fentanyl, hydromorphone, morphine, and oxycodone; while weak opioids included codeine, meperidine, pentazocine, propoxyphene, tapentadol, and tramadol. The duration of action was determined according to the galenic form: tablets, elixirs, syrups, injectable, oral solutions, and suppositories were classified as short-acting, while long-acting (12 h or 24 h) tablets, long-acting (12 h or 24 h) capsules and transdermal patches were classified as long-acting.

### Categorization of daily dose

For each opioid dispensation, the total dosage of opioids dispensed were calculated by multiplying the total number of tablets by the strength (dose) of the pills. Doses were converted to morphine equivalent daily dose (MEDD), according to established conversion guidelines, and categorized into less than 50 MEDD, between 50 and 100 MEDD, and greater than 100 MEDD [29, 36, 37]. These categories of daily dose have been used in previous studies [25, 29]. If a pregnancy had several dispensations of the same opioid but with different MEDD, the pregnancy was classified in the category with the highest MEDD. Conversion factors for mg of opioids to MEDD are shown in Table S1B in Additional file 1.

### Duration of cumulative exposure during pregnancy

The cumulative duration of opioid exposure during pregnancy was estimated by adding the durations for each opioid prescription that were filled and recorded in pharmacy claims database. This analysis was performed on all pregnancies regardless of pregnancy outcomes (*n* = 20,921). If a pregnancy had multiple dispensations of the same drug, the treatment duration was the total of the duration in each dispensation. If a pregnancy was exposed to more than one opioids, we included the overlap period of different opioid only once. We categorized the cumulative duration into less than 90 days or 90 days or more, which was considered as chronic opioid use during pregnancy [38].

### Patterns of use

To compare the duration of treatment between strong and weak opioid users, we defined 8 mutually exclusive categories considering cumulative duration (< 90 vs. ≥90 days), duration of action (short vs. long-acting) and strength of the opioid (weak vs. strong).

1) cumulative duration < 90 days: only weak, short-acting opioids;

2) cumulative duration < 90 days: ≥1 strong, short-acting opioid;

3) cumulative duration < 90 days: only weak, ≥1 long-acting opioid;

4) cumulative duration < 90 days: ≥1 strong, ≥1 long-acting opioid;

5) cumulative duration ≥90 days: only weak, short-acting opioids;

6) cumulative duration ≥90 days: ≥1 strong, short-acting opioid;

7) cumulative duration ≥90 days: only weak, ≥1 long-acting opioid;

8) cumulative duration ≥90 days: ≥1 strong, ≥1 long-acting opioid.

### Potential risk factors associated with opioid use during pregnancy

We considered the following variables as potential risk factors associated with opioid use during pregnancy: 1) socio-demographic variables measured on LMP (QPC data on gestational age have been validated [11]): maternal age, area of residence (urban/rural), and Quebec welfare recipients (low-income families receiving financial assistance); 2) maternal chronic comorbidities measured in the year prior to LMP: cancer, hypertension, asthma, epilepsy, depression, diabetes, autoimmune rheumatic diseases, drug dependence, alcohol dependence, and nicotine dependence; 3) health care utilization in the year prior to LMP: number of physician visits (general practitioner and specialist), hospitalization or emergency room visit (yes/no), and the use of other medications excluding study medication and medication used to identify comorbidities; 4) benzodiazepine use in the year prior to LMP.

Diagnostic and medication codes used to identify comorbidities are listed in Table S2 in Addition file 1**.**

### Descriptions of conditions associated with opioid use

For each pregnancy, we first identified all different prescribers of opioids during pregnancy, and then collected all medical visits to these prescribers in the 3-months period prior to the date of the prescription being filled and the associated ICD-9 and ICD-10 diagnostic codes. All the diagnostic codes for all exposed pregnancies were selected and ranked in the order of frequency.

Diagnostic codes associated with pain were considered as conditions associated with opioid use. The 3 months prior to the dispensation time-window was based on previous study [37]. Conditions associated with opioid use for users with < 90 days exposure vs ≥90 days exposure will be compared.

### Statistical analysis

Descriptive analyses were performed to summarize the characteristics of the study population; using chi-square and t-test for categorical and continuous variables, respectively. Annual prevalence of maternal opioids use was calculated from 1998 to 2015 for all pregnancies according to pregnancy outcome (delivery, planned abortion, and spontaneous abortion), trimester of exposure, individual opioids, and MEDD. Relative prevalence differences were calculated between years. To do this, the prevalence differences between calendar years were calculated by dividing the absolute prevalence difference between year A and year B by the prevalence of the year A, multiplied by 100%. The Cochran-Armitage test was used to test whether the prevalence over time had a significant linear trend. Mean and standard deviations of cumulative duration of exposure during pregnancy were calculated using descriptive analyses (Student’s t-test), according to the 8 categories of patterns of use. We compared durations of treatments between groups (use with long-acting opioids for more than 3 months, use of short-acting for more than 3 months, use with long-acting for less than 3 months, use of short-acting for than less 3 months) using ANOVA models. Head-to-head comparisons were done with Tukey analysis. Multivariate logistic regression models were used to identify risk factors associated with opioid use during pregnancy. All known potential risk factors for opioids use were entered into the multivariate generalized estimation equation models at once and adjusted for each other. All analyses were performed with SAS software (SAS Institute Inc.) version 9.4.

## Results

Of the 442,079 eligible pregnancies (262,125 women) within the QPC between 1998 and 2015, we identified 20,921 (4.7%) pregnancies exposed to prescribed opioids; and the prevalence increased by 41.6%, from 3.6% in 1998 and 5.1% in 2011, then stabilized at 4.9% in 2015 (Fig. S1 in Addition file 1).

**Fig. 1:**
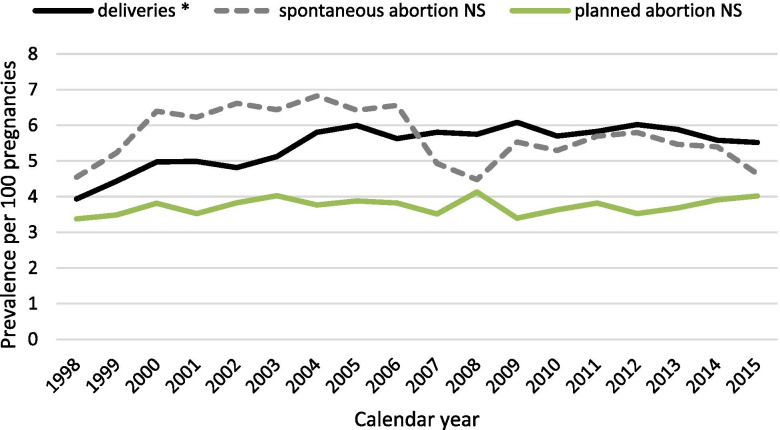
Prevalence of opioid exposure during pregnancy: by pregnancy outcome during the study period - 1998 to 2015. * trend test *p* < 0.001. NS: non-significant trend test

When looking at pregnancy outcomes, we observed that 5.4% of pregnancies ending with delivery were exposed to prescribed opioids, while 5.6% of spontaneous abortions (*n* = 160,867) and 3.7% of pregnancies ending in planned abortions (*n* = 31,978) were exposed to prescribed opioids. For pregnancies ending with deliveries, the prevalence of opioid exposed pregnancies increased significantly by 40.3%, from 3.9% in 1998 to 5.5% in 2015, while no significant change was observed for pregnancies ending in spontaneous and planned abortions (Fig. 1).

**Fig. 2:**
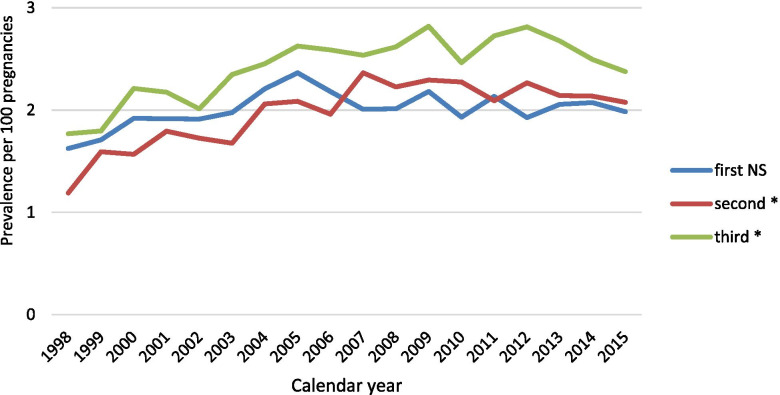
Prevalence of opioid exposure during pregnancy among pregnancy ending with a delivery by trimester of pregnancy during the study period - 1998 to 2015. * trend test *p* < 0.001. NS: non-significant trend test

Of the 249,234 pregnancies ending with delivery, the overall prevalence of opioid exposed pregnancies was 5.4%, with a prevalence of 2.0% in both the 1st trimester and the 2nd trimester, and 2.4% in the 3rd trimester. Notably, the prevalence of 2nd (1.19 to 2.08%) and 3rd (1.77 to 2.38%) trimesters opioid exposure increased significantly between 1998 and 2015 (*P* < 0.001). We did not observe a statistically significant increase over time for the first trimester (Fig. 2).

### Opioid use trends

Codeine was the most commonly dispensed opioid, accounting for 70% of all dispensed opioids (*n* = 22,847), followed by hydromorphone (11%), morphine (10%), oxycodone (5%), and meperidine (3%). Fentanyl, pentazocine, and tramadol represented less than 1% of dispensed opioids; no data were reported for propoxyphene and tapentadol. Strong opioids accounted for 26% of all opioid prescription fillings, while weak opioids accounted for 74% of all opioid prescription fillings. Over time, the prevalence of codeine exposed pregnancies increased by 29%, from 3.35% in 1998 to 4.33% in 2005, then decreased by 47%, from 4.33% in 2005 to 2.30% in 2015. The decrease in the prevalence of codeine exposed pregnancies was accompanied by increase in the prevalence of pregnancies exposed to morphine (48-fold increase, from 0.029 to 1.41%), hydromorphone (8.4-fold increase, from 0.115 to 1.08%), and oxycodone (19.6-fold increase, from 0.022 to 0.44%) from 1998 to 2015, and mostly for daily doses < 50 MEDD (Table 1 and Table S3).

**Table 1:** Prevalence of overall and individual opioids use during pregnancy among pregnancies ending with deliveries (*n* = 249,234), 1998–2015

year	Overall (%)	Codeine		Morphine		Hydromorphone		Oxycodone	
		%	% of overall	%	% of overall	%	% of overall	%	% of overall
1998	3.93	3.35	85.09	0.029	0.73	0.11	2.92	0.022	0.55
1999	4.43	3.69	83.11	0.034	0.78	0.18	4.03	0.044	0.99
2000	4.97	4.12	82.90	0.032	0.64	0.22	4.43	0.038	0.77
2001	4.99	3.94	78.99	0.061	1.23	0.31	6.27	0.045	0.90
2002	4.81	3.89	80.78	0.092	1.91	0.42	8.78	0.086	1.78
2003	5.12	3.92	76.49	0.140	2.73	0.58	11.28	0.109	2.12
2004	5.80	4.25	73.18	0.185	3.19	0.56	9.57	0.176	3.03
2005	5.99	4.33	72.31	0.300	5.01	0.59	9.78	0.217	3.62
2006	5.63	4.06	72.08	0.342	6.08	0.57	10.21	0.263	4.68
2007	5.81	3.73	64.31	0.367	6.32	0.66	11.34	0.308	5.30
2008	5.75	3.83	66.55	0.430	7.47	0.71	12.39	0.417	7.26
2009	6.08	3.89	63.97	0.595	9.78	0.66	10.88	0.376	6.18
2010	5.70	3.45	60.55	0.685	12.02	0.62	10.92	0.361	6.33
2011	5.83	3.37	57.73	0.935	16.03	0.66	11.25	0.543	9.31
2012	6.02	3.22	53.42	1.061	17.62	0.77	12.76	0.467	7.75
2013	5.89	2.90	49.27	1.198	20.36	0.89	15.04	0.425	7.21
2014	5.58	2.57	46.06	1.407	25.22	0.86	15.30	0.482	8.65
2015	5.52	2.30	41.71	1.408	25.50	1.08	19.49	0.444	8.05

Of the 22,847 different opioid prescriptions filled to 20,921 pregnancies, 20,481 prescriptions (90%) were filled for a daily dose of < 50 MEDD, 2084 (9%) for a daily dose 50 to 100 MEDD and 282 (1%) for a dose > 100 MEDD (Fig. S2 in Addition file 1).

### Opioid substitutes trends

We identified a total of 459 pregnancies that were exposed to opioid substitutes between 1998 and 2015, with no significant increase in the overall use during pregnancy over time. These 459 pregnancies resulted in 208 deliveries, 215 planned abortions and 36 spontaneous abortions. Among these 459 pregnancies, 423 (92%) pregnancies were exposed in the 1st trimester, 324 (85%) in the 2nd trimester (among 379 pregnancies longer than 15 weeks), and 176 (84%) in the 3rd trimester (among 209 pregnancies longer than 26 weeks). Overall, 437 were exposed to methadone, 24 to buprenorphine-naloxone, and two pregnancies were exposed to both substitutes.

### Patterns of opioid use and duration of treatment (Fig. 3)

Among all opioid exposed pregnancies, 92% (*n* = 19,194) were exposed to only one type of opioid, while 7% (*n* = 1556) to 2 types of opioids, and 1% (*n* = 171) to 3 or more different types of opioids.

**Fig. 3:**
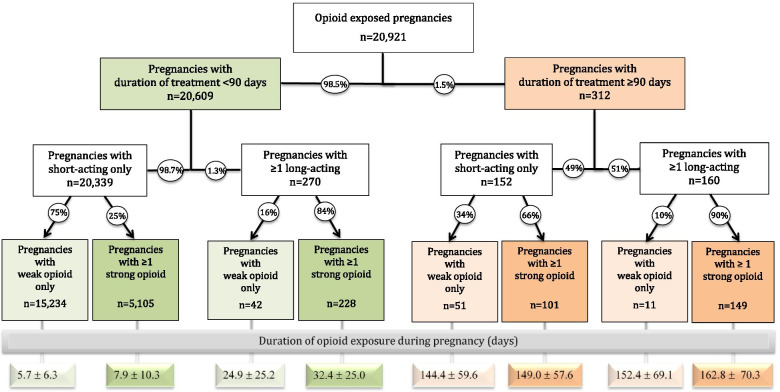
Patterns of opioids use during pregnancy by duration of treatment, duration of action and strength of the opioid medication. Strong opioids included fentanyl, hydromorphone, morphine and oxycodone. Weak opioids included codeine, meperidine, pentazocine, propoxyphene, tapentadol and tramadol

Of the 22,847 different opioids filled, 19,603 (85.8%) dispensed only one type of opioid, 2044 (9%) dispensed two types of opioids, and 1200 (5.2%) dispensed three or more types of opioids.

Exposure for a duration < 90 days represented 98.5% of all opioid exposed pregnancies and were mainly exposed to a short-acting weak opioid (74%). Overall 1.5% (*n* = 312) pregnancies were exposed to opioid during pregnancy for a duration ≥90 days, with 48% (*n* = 149) of them being on long-acting strong opioids.

For the 8 categories of opioid exposure, the average duration of opioid treatment during pregnancy were significantly longer among pregnancies exposed to strong opioids as compared to weak opioids regardless of the duration of treatment (< 90 days vs. ≥90 days) or the duration of action of the opioid type (long vs. short-acting) (*P* < 0.05) (Fig. 3).

### Conditions associated with opioids prescription fillings

Pregnancies exposed to opioids were more likely to be among women who were living in rural area, being a Quebec welfare recipient, having comorbidities (namely cancer, hypertension, asthma, epilepsy, depression, nicotine addiction), having used a benzodiazepine, having other prescribed medications, having ≥1 emergency room visit or hospitalization and having a general practitioner visit in the 12-months period before the LMP (Table 2). Pregnancies exposed to opioids were less common among women with alcohol dependence than women who did not have such issue.

**Table 2:** Potential risk factors associated with opioid use during pregnancy

	Number of pregnancies		OR (95% CI)	
	Opioid exposed *n* = 20,921	Opioid unexposed *n* = 421,158	Crude	Adjusted*
Maternal characteristics on first day of gestation, n (%)				
Age (year) – mean ± SD	27.7 ± 6.0	28.1 ± 6.1	0.99 (0.99–0.99)	0.99 (0.99–1.00)
Rural (vs. urban) dweller	3611 (17.3)	64,529 (15.3)	1.15 (1.10–1.19)	**1.17 (1.12–1.21)**
Welfare recipient	8050 (38.5)	106,719 (25.3)	1.79 (1.74–1.85)	**1.40 (1.35–1.44)**
Maternal comorbidities in the 12 months prior to the LMP, n (%)				
Cancer	227 (1.1)	2505 (0.6)	1.77 (1.53–2.04)	**1.30 (1.12–1.51**)
Autoimmune rheumatic diseases	147 (0.7)	1393 (0.3)	2.08 (1.74–2.49)	1.16 (0.96–1.40)
Hypertension	945 (4.5)	10,207 (2.4)	1.82 (1.69–1.96)	**1.12 (1.03–1.21)**
Diabetes	659 (3.1)	7564 (1.8)	1.71 (1.57–1.86)	1.01 (0.92–1.11)
Asthma	4238 (20.3)	44,886 (10.7)	2.01 (1.93–2.09)	**1.17 (1.12–1.22)**
Epilepsy	893 (4.3)	5781 (1.4)	3.05 (2.81–3.30)	**1.43 (1.31–1.56)**
Depression	4541 (21.7)	44,434 (10.5)	2.22 (2.14–2.31)	**1.28 (1.23–1.34)**
Benzodiazepine use	2883 (13.8)	22,663 (5.4)	2.60 (2.47–2.80)	**1.30 (1.23–1.37)**
Drug dependence	346 (1.6)	2517 (0.6)	2.47 (2.17–2.80)	0.93 (0.81–1.06)
Alcohol dependence	215 (1.0)	2002 (0.5)	1.92 (1.63–2.25)	**0.74 (0.63–0.88)**
Nicotine dependence	1734 (8.3)	15,583 (3.7)	2.19 (2.07–2.32)	**1.34 (1.26–1.42)**
Health services utilization in the 12 months prior to the LMP, n (%)				
Number of general practitioner visits – mean ± SD	7.8 ± 9.6	4.6 ± 5.9	1.05 (1.05–1.05)	**1.01 (1.01–1.02)**
Number of specialist visits – mean ± SD	5.6 ± 11.0	3.5 ± 6.7	1.02 (1.02–1.03)	1.00 (1.00–1.00)
Hospitalizations/emergency department visits (yes)	10,428 (49.8)	14,2801 (33.9)	1.83 (1.78–1.89)	**1.27 (1.23–1.31)**
Number of comedications** - mean ± SD	3.8 ± 3.5	2.1 ± 2.4	1.20 (1.19–1.20)	**1.12 (1.12–1.13)**

*Adjusted for all variables included in this table

*SD* standard deviation; *LMP* last menstrual period

**Number of other comedications dispensed, excluding opioids, antihypertensive drugs, antidiabetic drugs, asthma medications, medications for epilepsy, antidepressants and benzodiazepines

Of all opioid exposed pregnancies, 71.1% (*n* = 14,883) had ≥1 visit to the opioid prescriber with an associated ICD-9 and ICD-10 diagnostic codes in the 3-months period before the date of the prescription fill. The most common diagnosis was abdominal pain (18.1%), followed by musculoskeletal pain (17.2%), headache (5.8%), dental pain (3.4%), chest pain (1.7%), ear pain (1.6%), and neuropathic pain (1.0%) (Table 3). Pregnancies exposed to opioid for ≥90 days were more likely to have musculoskeletal pain and neuropathic pain than pregnancies exposed to opioid for < 90 days.

**Table 3:** Conditions associated with opioid fillings among pregnancies with at least one diagnosis in the previous three months (*n* = 14,883)

Pain, etiology	Overall (%)	Used < 90 days (%)	Used ≥90 days (%)
Abdominal pain	**2696 (18.1)**	**2640 (18.1)**	**56 (20.6)**
Urinary tract infection	436 (2.9)	422 (2.9)	14 (5.1)
Gynecologic origin	256 (1.7)	245 (1.7)	11 (4.0)
Obstruction of urinary tract	415 (2.8)	412 (2.8)	3 (1.1)
Calculus of bile duct	222 (1.5)	221 (1.5)	1 (0.4)
Oesophageal and stomach disease	86 (0.6)	76 (0.5)	10 (3.7)
Gastroenteritis and colitis	75 (0.5)	73 (0.5)	2 (0.7)
Appendicitis	73 (0.5)	73 (0.5)	0 (0.0)
Musculoskeletal pain	**2564 (17.2)**	**2423 (16.6)**	**141 (51.8)**
Lumbar disc origin	909 (6.1)	846 (5.8)	63 (23.2)
Dislocation sprain	577 (3.9)	551 (3.8)	26 (9.6)
Fracture	249 (1.7)	247 (1.7)	2 (0.7)
Neck pain	80 (0.5)	73 (0.5)	7 (2.6)
Auto-immune rheumatic disease	22 (0.1)	14 (0.1)	8 (2.9)
Headache	**862 (5.8)**	**839 (5.7)**	**23 (8.5)**
Migraine	302 (2.0)	291 (2.0)	11 (4.0)
Sinusitis	277 (1.9)	269 (1.8)	8 (2.9)
Dental pain	**502 (3.4)**	**497 (3.4)**	**5 (1.8)**
Chest pain	**256 (1.7)**	**250 (1.7)**	**6 (2.2)**
Ear pain	**245 (1.6)**	**244 (1.7)**	**1 (0.4)**
Neuropathic pain	**157 (1.0)**	**143 (1.0)**	**14 (5.1)**
Other pain	**1007 (6.8)**	**986 (6.7)**	**21 (7.7)**
Cellulitis and skin abscess	650 (4.4)	637 (4.4)	13 (4.8)
Cancer	33 (0.2)	32 (0.2)	1 (0.4)
Wounds Bruises	324 (2.2)	317 (2.2)	7 (2.6)
Others conditions	**7645 (51.4)**	**7565 (51.8)**	**80 (29.4)**

## Discussion

Within the QPC, we observed a 40.3% increase in the prevalence of opioid use having gone from 3.9% in 1998 to 5.5% in 2015; specifically, in the second (1.19% in 1998 to 2.08% in 2015) and third (1.77% in 1998 to 2.38% in 2015) trimesters of pregnancy. We analyzed the duration of opioid use according to 8 categories. The average duration of opioid use was significantly longer among pregnancies exposed to strong opioids as compared to weak opioids regardless of the duration of treatment (< 90 vs. ≥90 days) or the duration of action of the opioid (short vs. long-acting). To our knowledge, this finding is novel. Among the 442,079 eligible pregnancies, 20,921 (4.7%) were exposed to prescribed opioids. Codeine was the most commonly dispensed opioids (70% of all dispensed opioids), however the prevalence of its’ use decreased by 47%, from 4.3% in 2005 to 2.3% in 2015. This decrease was accompanied by an increased use of strong opioids between 1998 and 2015, namely morphine (48-fold increase), hydromorphone (8.4-fold increase) and oxycodone (19.6-fold increase).

Falk et al. [8] reported a modest increase in opioid use during pregnancy from 7.3% in 2001 to 7.7% in 2013 in Manitoba while the mean opioid use per pregnancy, measured as morphine equivalents, increased 4.3-fold over the study period.

It should be noted that replacing first-line opioid codeine with a second-line strong opioid such as morphine, hydromorphone and oxycodone, particularly during pregnancy, goes against the guidelines for safe and effective use of opioids in the treatment of chronic noncancer pain [25, 39].

Based on these guidelines, when needed, less potent opioids should be used first [40–42], as codeine has lower rates of overdose, misuse and addiction than more potent opioids [25–28]. Additionally, second-line opioids such as oxycodone, morphine and hydromorphone are not particularly responsive to chronic noncancer pain, and there is increasing evidence for the limited role of these opioids to treat chronic noncancer pain [25, 40–44].

In the context of pregnancy, associations between prenatal opioid exposure and neonatal abstinence syndrome [16–19], as well as certain congenital malformations [10, 20, 21, 45] have been reported in recent studies. Bateman et al. [24], using the Medicaid Analytic eXtract (2000–2014) and MarketScan (2003–2015) databases, have shown an increased risk of oral clefts associated with hydrocodone (RR 1.39, 95%CI 1.06–1.83) and oxycodone (RR 1.34, 95%CI 0.72–2.50) use in early pregnancy, but not with codeine (RR 0.74, 95%CI 0.40–1.37, 11 exposed cases), which is a weaker opioid. Furthermore, Wen et al. [46], using Optum’s deidentified Clinformatics Data Mart (2010–2017), have shown an increased risk of neurodevelopmental disorders in early childhood born to women receiving prescription opioids for longer duration, ≥14 days, (HR 1.70, 95%CI 1.05–2.96) or high doses (HR 1.22, 95%CI 1.01–1.54). Particularly, hydrocodone (adjusted HR 1.33, 95%CI 1.04–1.70) use in pregnancy was associated with increased risk of neurodevelopmental disorders, but not with codeine (adjusted HR 0.93, 95%CI 0.68–1.28, 41 exposed cases).

The 2017 American College of Gynecology and Obstetrics opioids use in pregnancy guidelines emphasize the need to avoid or minimize opioids use for pain management [27]. The 2017 Canadian guidelines for chronic noncancer pain emphasizes nonopioid treatment, and restricts the prescribed opioid dose to < 90 mg MEDD [28].

Our overall prevalence of 5.4% of opioid exposure was more consistent with recent studies. Wen et al. [12] reported 7.5% of opioid exposure among Rhode Island Medicaid covered women during pregnancy; the prevalence of prescribed opioids during pregnancy increased from 4.9% in 2008 to 11.1% in 2015 [12]. More importantly, strong opioids, hydrocodone and oxycodone were the most commonly dispensed opioids (66–82% of all prescribed opioids from 2008 to 2015) [12].

Elliott et al. [5] reported 7.5% of opioid exposure among Upper Midwest commercially and Medicaid-insured women during pregnancy from 2006 to 2014, with a significant decrease overtime (9% in 2006 to 6% in 2014) [5]. Oxycodone (54%) and hydrocodone (26% of all prescribed opioids) were the most commonly dispensed opioids compounds.

The difference in the prevalence of opioid use between studies may relate to variations in the characteristics of the studied populations, as well as the differences in health provider’s prescribing practice [5]. Overprescribing practices appear to be the force driving the opioid epidemic [22]. As a drug with addictive potential, opioid should be reserved for persistent pain despite non-narcotic treatments such as in terminal cancer patient, second degree burns, and major surgery [47]. However, patient satisfaction and pain-free expectations have resulted in opioids being prescribed for common pain conditions encountered in pregnancy [41]. This has led to the current opioid epidemic.

As early as 2005, HealthPartners Medical Group (HPMG), an large integrated health care system in the United States [5], implemented key strategies encouraging opioids providers for more careful assessments and offer nonopioid and nonpharmaceutical treatment strategies for pain in women of reproductive age, these approaches allowed them to counter the national trend, decreasing the opioid prescription rate over time [5], which imply that unnecessary prescribing and use of opioids among pregnant women can be reduced through targeted interventions and communication strategies.

In our cohort, the most frequent conditions associated with opioid use during pregnancy was abdominal pain, musculoskeletal pain, and headache, which is consistent with other studies [4, 5, 12]. Similar to our findings, recent studies show a rapid growth in pregnant women using high potency opioids for common pain management during their pregnancies [3, 4, 6]. However, opioids are not the preferred treatments for these conditions [27, 28], as they are not particularly opioid responsive and are manageable with other nonopioids medications, and nonpharmacological therapies, such as physical or psychotherapy [41]. Given the differences in the safety profile between strong opioids and the major weak opioid codeine [24, 46], the increased use of strong opioids during pregnancy with longer treatment duration raises public health concerns.

### Strength and limitation

Study strengths include the use of a population-based prospective pregnancy cohort with linkage of data at the individual level, which minimized selection and recall biases; this also allowed for analyses on a large number of pregnancies with detailed information regarding exposure, outcomes, and potential confounders. The QPC data on prescriptions filled [33] and gestational age [32] have been validated.

Since the QPC database only include women covered by the Quebec Public Prescription Drug Insurance, generalizability of results to those insured privately could be affected. However, validation studies have shown that publicly insured pregnant women have similar characteristics and co-morbidities with those who have private medication insurance [35]. The number of pregnancies exposed to opioids could be underestimated, as the QPC does not include opioid administration during hospital stays, weak opioid purchases without prescriptions, and do not capture the use of illicitly obtained drugs on the black-market. We considered filled prescriptions and not actual intake, but we have shown that prescription filling data in the QPC were valid when compared to maternal report [33]. Nevertheless, given that opioids are used on an as-needed basis, misclassification of exposure can remain especially in filled prescriptions before LMP. Looking at predictors of dichotomous opioids use is an important step in determining the characteristics of users, but can be too unspecific. We are missing data on prior opioid use which could be a predictor/determinant of use during pregnancy. Excluding pregnancies ending in miscarriage or abortion from the trimester calculation may underestimate the prevalent of prescribed opioids use during pregnancy, particularly the first trimester of pregnancy, as vast majority of miscarriages or abortions occur at first trimester of pregnancy. Although the study spanned for a 17-year period, it ended in 2015, which could not entirely represent the current situation of opioid exposure patterns in pregnancy. Group-based trajectory model has been increasingly used in the study of prescription medications [48–51], as the Group-based trajectory modelling enables comprehensive analysis of adherence over time [49, 50]. Given that our data on prescription fillings do not allow us to determine adherence per se, especially for medications that are used on an as-needed basis such as in the case for opioids, grouping categories of filled opioids prescriptions were chosen to better explain findings for clinical settings. In our study, alcohol dependence was identified in the RAMQ medical file (outpatient diagnoses, medical procedures) and MedEcho databases (in-hospital diagnoses and procedures) and defined according to ICD-9 and ICD-10 codes. Previous studies [5, 12, 52] have shown that pregnant women with alcohol dependence were more likely to be using opioids during pregnancy, which is the opposite to what has been seen for alcohol in our study. Given the nature of our data however, misclassification (underestimation) of alcohol dependence is possible, and would therefore lead to underestimation of the association between alcohol dependence and opioids use in our study. We did not do a sensitivity analysis to address the issue of multiple pregnancies within the same women. However, we have taken multiple pregnancies per women into account using generalized estimation equation models, which considers family clustering (multiple pregnancies per women). Furthermore, using the US national data (2005 to 2011), among privately insured pregnant women, Bateman et al. [3] have shown a similar frequency of exposure in first pregnancies (14.4%), second pregnancies (14.1%), or third or greater pregnancies (14.9%) within the same women, which is reassuring.

### Conclusions

In this population-based cohort, among publicly insured pregnant women, we observed a 40.3% increase in the prevalence of opioid use during pregnancy, from 3.9% in 1998 to 5.5% in 2015. The average durations of opioid exposure were significantly longer among pregnancies exposed to strong opioid as compared to weak opioids regardless of the cumulative duration of opioid treatment or the duration of action. Given the differences in the safety profile between strong opioids and the major weak opioid codeine, our study raises public health concerns.

## Supplementary Information

**Additional file 1:.**

## Data Availability

All data generated or analysed during this study are included in this published article and its supplementary information files.

## Abbreviations

MEQ: Mean morphine equivalents
QPC: Quebec Pregnancy Cohort
LMP: First day of the last menstrual period
RAMQ: Quebec medical claims database
MedEcho: Quebec hospitalization archives databases
ISQ: Quebec Statistics database
MEDD: Morphine equivalent daily dose
ICD-9: International Classification of Diseases, Ninth Revision
ICD-10: International Classification of Diseases, Tenth Revision
