# Supplementary material for: Prevalence and duration of prescribed opioid use during pregnancy: a cohort study from the Quebec Pregnancy Cohort
Source: BMC Pregnancy Childbirth. 2021 Dec 1;21:800. doi: 10.1186/s12884-021-04270-x (PMC8638412; doi:10.1186/s12884-021-04270-x)
Supplement: Supplementary file 1 — Additional file 1:. [file 12884_2021_4270_MOESM1_ESM.docx]

**Additional file 1. Supplemental tables and figures**

This additional file has been provided by the authors to give readers additional information about their work.

Supplement to: “Prevalence and duration of prescribed opioid use during pregnancy: a cohort study from the Quebec Pregnancy Cohort” By Jin-Ping Zhao, Christelle Berthod, Odile Sheehy, Behrouz Kassaï, Jessica Gorgui, Anick Bérard.

**Content:**

**Fig. S1** Prevalence of opioid exposure during pregnancy by calendar year.

**Fig. S2**. Prevalence of opioid exposure during pregnancy by molecules and by morphine equivalent daily dose (MEDD) categories during the study period - 1998 to 2015.

**Table S1A.** List of Opioid molecules.

**Table S1B.** Conversion factor of Morphine Equivalent Daily Dose (MEDD).

**Table S2.** List of diagnostic codes (ICD-9 and ICD-10) and medications used for the comorbidities**.**

**Table S3.** Prevalence of overall and individual opioids use during pregnancy among pregnancies ending with deliveries (n=249,234), 1998-2015

**Fig. S1** Prevalence of opioid analgesic exposure during pregnancy by calendar year

The prevalence of prescribed opioids use increased by 41.6% (p<0.001) between 1998 and 2011 and then stabilized.

**Fig. S2** Prevalence of opioid exposure during pregnancy by molecules and by morphine equivalent daily dose (MEDD) categories during the study period - 1998 to 2015

MEDD: Morphine equivalent daily dose

**Table S1A.** List of Opioid molecules

Medication generic codes:

| **Generic name** | **Quebec generic code** |
| --- | --- |
| Codeine | 2119, 17641, 38184, 38496, 46013, 46098, 46368, 46372, 46871, 47155 |
| Meperidine | 5603, 46412, 46168, 46172 |
| Pentazocine | 44528 |
| Tapentadol | 47860 |
| Propoxyphene | 2678, 38132,42149, 46198 |
| Tramadol | 47654, 47567 |
|  |  |
| Morphine | 6305, 19063, 43527, 44541 |
| Hydromorphone | 4615, 17771, 46790 |
| Oxycodone | 6799, 46036, 46037, 46344, 46651, 47846, 47908 |
| Fentanyl | 3809, 33855, 46478, 47038 |

| **Table S1B. Conversion factor of Morphine Equivalent Daily Dose (MEDD)** | |
| --- | --- |
| **Generic name of opioid analgesics** | **Conversion factor** |
| Codeine | 0.15 |
| Meperidine | 0.10 |
| Pentazocine | 0.37 |
| Tramadol | 0.10 |
| Morphine | 1.0 |
| Hydromorphone | 4.0 |
| Oxycodone | 1.5 |
| Fentanyl | 2.4 |

**Table S2. List of diagnostic codes (ICD-9 and ICD-10) and medications used for the comorbidities.**

**Hypertension**

ICD-9 codes: 401.0-405.9, 642.0-642.9 and 796.2

ICD-10 codes: I10.0, I10.1, I15.0, I15.1, I15.2, I15.8, I15.9, O10, O11, O12, O13, O14, O15 and O16

Medication generic codes:

| **Generic name** | **Quebec generic code** |
| --- | --- |
| Clonidine | 10751 |
| Methyldopa | 6136 |
| Hydralazine | 4524 |
| Minoxidil | 41564 |
| Doxazosine | 45625 |
| Prazosin | 37742 |
| Terazosin | 45520 |
| Acebutolol | 45463 |
| Atenolol | 43670 – 46325 -46315 |
| Bisoprolol | 47355 |
| Carvedilol | 47199 - 46319 |
| Labetalol | 45243 |
| Metoprolol | 38275 – 46763 - 46780 |
| Nadolol | 40563 |
| Oxprenolol | 42162 |
| Pindolol | 39016 |
| Pindolol-HCTZ | 45408 |
| Propranolol | 8229 |
| Sotalol | 44866 |
| Timolol | 38314 |
| Amlodipine | 47006 |
| Amlodipine/Atorvastatine |  |
| Felodipine | 45624 |
| Nifedipine | 42708 – 46388 - 46469 |
| Nifedipine-AAS | 47751 |
| Nimodipine | 45532 |
| Diltiazem | 43228 - 47247 |
| Verapamil | 40550 - 46573 |
| Verapamil-Trandolapril | 47440 |
| Benazepril | 47049 |
| Captopril | 42071 |
| Cilazapril | 47056 |
| Cilazapril-HCTZ | 47320 |
| Enalapril | 45476 |
| Enalapril-HCTZ | 45572 |
| Fosinopril | 47002 |
| Lisinopril | 45576 |
| Lisinopril-HCTZ | 47040 |
| Perindopril | 47117 - 46258 |
| Perindopril-Indapamide | 47449 |
| Quinapril | 45629 |
| Quinapril-HCTZ | 47301 |
| Ramipril | 47079 - 46216 |
| Ramipril-HCTZ | 47655 |
| Trandolapril | 47250 |
| Trandolapril/Verapamil |  |
| Candesartan | 46529 - 47309 |
| Candesartan-HCTZ | 46760 - 47412 |
| Eprosartan | 47389 |
| Eprosartan-HCTZ | 47534 - 47532 |
| Irbesartan | 46459 - 47282 |
| Irbesartan-HCTZ | 47354 |
| Losartan | 47135 – 46284 - 46441 |
| Losartan-HCTZ | 47207 |
| Olmesartan medoxomil | 47763 |
| Olmesartan medoxomil-HCTZ | 47764 |
| Telmisartan | 47333 - 46587 |
| Telmisartan-HCTZ | 47413 |
| Telmisartan/Amlodipine | N.A. |
| Valsartan | 46418 - 47259 |
| Valsartan-HCTZ | 47369 |
| Spironolactone | 9100 - 46572 |
| Ethacrynique | 3562 |
| Furosemide | 4173 |
| Amiloride | 41759 |
| Amiloride-HCTZ | 41772 |
| Hydrochlorothiazide | 4537 |
| Chlorthalidone | 1976 |
| Indapamide | 43397 |
| Metolazone | 19440 |
| Amiloride-HCTZ | 41772 |
| Spironolactone-HCTZ | 38158 |
| Triamtérène-HCTZ | 38197 |
| Triamtérène | 9763 |
| Amlodipine-Atorvastatine | 47609 |
| Aliskirene | 47706 |
| Aliskirene-HCTZ | 47823 |
| Excluding the medication on the following formulation: | |
| Formulation | Code |
| Ophthalmic powder | 1479 |
| Ophthalmic ointment | 1624 |
| Ophthalmic solution | 2204 |
| Ophthalmic and Optic Solution | 2233 |
| Ophthalmic suspension | 2784 |
| Ophthalmic and Optic Suspension | 2813 |
| Ophthalmic Irrigation Solution | 3480 |
| Ophthalmic gel | 5365 |
| Ophthalmic Gel Solution | 5599 |

**Diabetes**

ICD-9 codes: 250.0-250.9, 271.4 and 790.2

ICD-10 codes: E10-E14 and R73.0

Medication generic codes:

| **Generic name** | **Quebec generic code** |
| --- | --- |
| Metformine | 5824 - 47208 |
| Glucagon | 4238 |
| Chlorpropamide | 1937 |
| Glyburide | 4264 |
| Tolbutamide | 9672 - 15184 |
| Gliclazide | 46056 - 47329 |
| Glimepiride | 46799 - 47427 |
| Acarbose | 46300 - 47151 |
| Pioglitazone | 46678 - 47392 |
| Rosiglitazone | 47371 - 46642 |
| Rosiglitazone/Metformine | 46862 |
| Rosiglitazone/Glimepiride | 47652 |
| Nateglinide | 46810 |
| Repaglinide | 47357 - 46568 |
| Saxagliptine | 47817 |
| Sitagliptine | 47715 |
| Sitagliptine/Metformine | 47807 - 47832 |
| Insuline aspart | 46798 - 47424 |
| Insuline aspart/ Insuline aspart protamine |  |
| Insuline glulisine | 47749 |
| Insuline isophane bio-synthétique | 44164 |
| Insuline lispro | 46322 - 47206 |
| Insluline zinc cristalline bio-synthétique | 44489 |
| Insulines zinc cristalline et isophane bio-synthétique | 45531 |
| Insuline aspart/insuline aspart protamine | 47615 |
| Insuline détémir | 47586 |
| Insuline glargine | 47536 |
| Insuline lispro/insuline lispro protamine | 47426 |
| Insuline globine zinc | 4823 |
| Insuline sulfatée | 4888 |
| Insuline zinc cristalline (porc) | 18296 |
| Insuline protamine zinc (boeuf) | 18309 |
| Insuline protamine zinc (porc) | 18322 |
| Insuline isophane (porc) | 18335 |
| Insuline isophane (boeuf) | 18348 |
| Insuline lente (boeuf et porc) | 39120 |
| Insuline isophane (boeuf et porc) | 39133 |
| Insuline protamine zinc (boeuf et porc) | 39146 |
| Insuline semilente (boeuf et porc) | 39159 |
| Insuline ultralente (boeuf et porc) | 39172 |
| Insuline zinc cristalline (boeuf et porc) | 39185 |
| Insuline isophane (boeuf) * | 39458 |
| Insuline protamine zinc (boeuf) * | 39484 |
| Insuline protamine zinc (porc) * | 39497 |
| Insuline zinc cristalline (boeuf) * | 39523 |
| Insuline lente (porc) | 41655 |
| Insuline zinc cristalline (porc)/ insuline isophane (porc) | 43033 |
| Insuline zinc cristalline (boeuf) | 43735 |
| Insuline isophane semi-synthétique de séquence humaine | 44151 |
| Insuline lente semi-synthétique de séquence humaine | 44476 |
| Insuline zinc cristalline semi-synthétique de séquence humaine | 44502 |
| Insuline ultralente semi-synthétique de séquence humaine | 44996 |
| Insulines isophane et zinc cristalline semi-synthétiques de séquence humaine | 45405 |
| Insuline lente bio-synthétique de séquence humaine | 45415 |
| Insuline ultralente bio-synthétique de séquence humaine | 45483 |
| Insulines isophane et zinc cristalline bio-synthétique de séquence humaine | 45511 |
| Insulines zinc cristalline et isophane semi-synthétiques de séquence humaine | 45534 |
| Insuline zinc cristalline (boeuf et porc) | 46536 |
| Insuline isophane (boeuf et porc) | 46537 |
| Insuline lente (boeuf et porc) | 46538 |
| Insuline isophane(humaine)/ insuline injectable(humaine) | 46592 |
| Insuline isophane (humaine) | 46602 |
| Insuline injectable (humaine) | 46603 |
| Insuline lispro/insuline isophane (humaine) | 46607 |
| Insuline zinc cristalline (porc) * | 47004 |
| Insuline lispro/ insuline lispro protamine | 47426 |
| Alogliptine |  |
| Alogliptine/Metformine |  |
| Canagliflozine |  |
| Dipagliflozine |  |
| Glidazide |  |
| Glimepiride |  |
| Linagliptine |  |
| Linagliptine/Metformine |  |
| Liragludine |  |

**Asthma**

ICD-9 codes : 493.0, 493.1, 493.3, 493.4, 493.5, 493.6, 493.7, 493.8 and 493.9

ICD-10 codes: J45.0, J45.1, J45.8 and J45.9

Medication generic codes

| **Generic name** | **Quebec generic code** |
| --- | --- |
| Aminophylline | 364, 46428 |
| Beclomethasone | 780* |
| Budesonide | 45499* |
| Budesonide/ formoterol | 47428, 46800 |
| Cromoglicate sodique | 39419, 47315 |
| Cromoglycate disodique | 2223 |
| Epinephrine | 3380 |
| Epinephrine | 3406 |
| Epinephrine racemic | 3419 |
| Fenoterol | 38548 |
| Flunisolide | 38730* |
| Fluticasone | 47050*,46435* |
| Formoterol | 47231* |
| Formoterol | 47271,46430 |
| Formoterol / budesonide | 47428 |
| Ipratropium (bromure d') | 43124, 46640 |
| Ipratropium (bromure d')/ salbutamol (sulfate de) | 47186, 46302 |
| Isoproterenol (chlorhydrate d') | 5083 |
| Isoproterenol (chlorhydrate d')/ phenylephrine (bitartrate de) | 5096 |
| Isoproterenol (chlorhydrate d')/ phenylephrine (chlorhydrate de) | 5109 |
| Isoproterenol (sulfate d') | 5070 |
| Ketotifene (fumarate de) | 45555, 46752 |
| Montelukast sodique | 47303, 47302, 46467 |
| Nedocromil sodique | 47033, 45563, 46463 |
| Orciprenaline (sulfate d') | 6721 |
| Oxtriphylline | 43475 |
| Pirbuterol (acetate de) | 47153,46299 |
| Procaterol hemihydrate (chlorhydrate de) | 45547 |
| Salbutamol | 10530 |
| Salbutamol (sulfate de) | 33634,46737 |
| Salmeterol (xinafoate de ) / fluticasone (propionate de ) | 47335,46597 |
| Salmeterol (xinafoate de) | 47112,46247 |
| Terbutaline (sulfate de) | 34180 |
| Theophylline | 9464,46847 |
| Theophylline (aminoacetate calcique de) | 9490 |
| Theophylline (aminoacetate sodique de) | 9503 |
| Theophylline/dextrose | 44944 |
| Triamcinolone (acetonide) | 9737* |
| Zafirlukast | 47266,46401 |
| Ciclesonide | 47626* |
| Momethasone | 45581 |
| Momethasone/Formeterol |  |
| Zolair |  |

For code with a * use only the following formulations:

| **Formulation** | **Code** |
| --- | --- |
| Powder aerosol | 1305 |
| Powder aerosol with applicator | 1334 |
| Aerosol solution | 1856 |
| Aerosol solution with applicator | 1885 |
| Solution for Inhalation | 1972 |
| Suspension aerosol | 2610 |
| Suspension aerosol with applicator | 2639 |
| Inhalation powder with applicator | 5563 |
| Inhalation powder | 5564 |
| Oral spray | 5584 |
| Gel | 5619 |
| Powder for solution for inhalation | 5634 |

**Depression**

ICD-9 codes: 296, 309, 311, and 300.4

ICD-10 codes: F30, F31, F32, F33, F34, F38 and F39.

Medication generic codes:

| Generic name | Quebec generic code |
| --- | --- |
| Citalopram | 46543 - 47317 |
| CEscitalopram | 47553 |
| Fluoxetine | 45504 |
| Fluvoxamine | 45633 |
| Paroxetine | 46164 – 47061 |
| Sertraline | 45630 |
| Viibryd | NA |
| Desvenlafaxine | NA |
| Duloxetine | 47714 |
| Milnacipran | NA |
| Venlafaxine | 46244 - 47118 |
| Isocarboxazid | 5018 |
| Phenelzine | 7280 |
| Tranylcypromine | 9698 |
| Amitriptyline | 442 – 429 - 46836 |
| Amoxapine | 43696 |
| Clomipramine | 14781 |
| Desipramine | 2522 |
| Doxepin | 3198 |
| Imipramine | 4784 |
| Maprotiline | 37443 |
| Nortriptyline | 46835 – 6578 |
| Protriptyline | 8294 |
| Trimipramine | 9906 |
| Bupropion | 46435 – 47285 |
| Buspirone | 45609 |
| Maprotiline | 37443 |
| Mirtazapine | 46744 – 47408 |
| Reboxetine | NA |
| Trazodone | 43137 |
| Vilazodone | NA |
| Moclobemide | 46427 – 47005 |
| L-tryptophane | 42058 |
| Nefazodone | 46235-47093 |

**Cancer**

ICD-9 codes: 140-209

ICD-10 codes: C00-C95

**Autoimmune rheumatic diseases**

ICD-9 codes: 710.0, 695.4, 714, 340, 709.1, 446.0, 447.5, 710.3, 710.1, 710.2, 136.1, 710.9

ICD-10 codes: M32, L93, M05, G35, L95, M30, M31, M33, M34, M35.0, M35.2, M35.9

**Tobacco dependence**

ICD-9 codes: 305.1

ICD-10 codes: F17.2, Z72.0 and T65.2

Medication generic codes:

| **Generic name** | **Quebec generic code** |
| --- | --- |
| nicotine (gums and patches) | 46093 |
|  | 47363 |
|  | 47364 |
|  | 46129 |
|  | 46339 |
|  | 46689 |

**Alcohol dependence and illnesses secondary to chronic alcohol use**

ICD-9 codes: 303.9, 291.0, 291.1, 291.2, 291.3, 291.8, 291.9, 571.1, 571.0, 571.2, 571.3, 980.0-980.9, 577.1, 255.0, 359.4, 425.5, 535.3, 655.4, 357.5

ICD-10 codes: F102-F109, K701-K703, K709, T510 -T519, K852, K860, E244, G721, I426, K292, O354, G621, O993

**Other drugs dependence**

ICD-9 codes: 304.0 and 304.9

ICD-10 codes: F11.2

**Benzodiazepine use**

Medication generic codes:

| **Generic name** | **Quebec generic code** |
| --- | --- |
| Alprazolam | 43501 |
| Bromazepam | 43488 |
| Chlordiazepoxide | 1807 |
| Clobazam | 45591 |
| Clonazepam | 37872 |
| Clorazepate | 14768 |
| Diazepam | 2717 |
| Diazepa | 46161 |
| Flurazepam | 4095 |
| Flurazepam | 46818 |
| Lorazepam | 37950 |
| Lorazepam | 46440 |
| Midazolam | 45492 |
| Nitrazepam | 42045 |
| Oxazepam | 6786 |
| Temazepam | 41590 |
| Triazolam | 39029 |
| Zopiclone | 46047 |
| Zaleplon | 46668 |
| Alprazolam | 43501 |
| Bromazepam | 43488 |
| Chlordiazepoxide | 1807 |

**Table S3.** Prevalence of overall and individual opioids use during pregnancy among pregnancies ending with deliveries (n=249,234), 1998-2015

| year | Overall (%) | Codeine | | Morphine | | Hydromorphone | | Oxycodone | | Meperidine | | Fentanyl | | Pentazocine | | Tramadol | |
| --- | --- | --- | --- | --- | --- | --- | --- | --- | --- | --- | --- | --- | --- | --- | --- | --- | --- |
|  |  | % | % overall | % | % overall | % | % overall | % | % overall | % | % overall | % | % overall | % | % overall | % | % overall |
| 1998 | 3.93 | 3.347 | 85.09 | 0.029 | 0.73 | 0.115 | 2.92 | 0.022 | 0.55 | 0.180 | 4.58 | 0 | 0.00 | 0.0431 | 1.10 | 0 | 0.00 |
| 1999 | 4.43 | 3.685 | 83.11 | 0.034 | 0.78 | 0.179 | 4.03 | 0.044 | 0.99 | 0.235 | 5.30 | 0 | 0.00 | 0.0125 | 0.28 | 0 | 0.00 |
| 2000 | 4.97 | 4.123 | 82.90 | 0.032 | 0.64 | 0.221 | 4.43 | 0.038 | 0.77 | 0.278 | 5.59 | 0 | 0.00 | 0.0128 | 0.26 | 0 | 0.00 |
| 2001 | 4.99 | 3.939 | 78.99 | 0.061 | 1.23 | 0.313 | 6.27 | 0.045 | 0.90 | 0.264 | 5.29 | 0.0032 | 0.06 | 0.0193 | 0.39 | 0 | 0.00 |
| 2002 | 4.81 | 3.888 | 80.78 | 0.092 | 1.91 | 0.423 | 8.78 | 0.086 | 1.78 | 0.248 | 5.16 | 0.0032 | 0.07 | 0.0064 | 0.13 | 0 | 0.00 |
| 2003 | 5.12 | 3.919 | 76.49 | 0.140 | 2.73 | 0.578 | 11.28 | 0.109 | 2.12 | 0.345 | 6.74 | 0.0078 | 0.15 | 0.0116 | 0.23 | 0 | 0.00 |
| 2004 | 5.80 | 4.246 | 73.18 | 0.185 | 3.19 | 0.555 | 9.57 | 0.176 | 3.03 | 0.242 | 4.17 | 0.0095 | 0.16 | 0.0095 | 0.16 | 0 | 0.00 |
| 2005 | 5.99 | 4.334 | 72.31 | 0.300 | 5.01 | 0.586 | 9.78 | 0.217 | 3.62 | 0.258 | 4.31 | 0.0277 | 0.46 | 0.0046 | 0.08 | 0 | 0.00 |
| 2006 | 5.63 | 4.056 | 72.08 | 0.342 | 6.08 | 0.574 | 10.21 | 0.263 | 4.68 | 0.202 | 3.59 | 0.0175 | 0.31 | 0 | 0.00 | 0 | 0.00 |
| 2007 | 5.81 | 3.733 | 64.31 | 0.367 | 6.32 | 0.658 | 11.34 | 0.308 | 5.30 | 0.236 | 4.06 | 0.0506 | 0.87 | 0.0084 | 0.14 | 0 | 0.00 |
| 2008 | 5.75 | 3.826 | 66.55 | 0.430 | 7.47 | 0.712 | 12.39 | 0.417 | 7.26 | 0.217 | 3.77 | 0.0041 | 0.07 | 0.0082 | 0.14 | 0 | 0.00 |
| 2009 | 6.08 | 3.891 | 63.97 | 0.595 | 9.78 | 0.662 | 10.88 | 0.376 | 6.18 | 0.179 | 2.94 | 0.0089 | 0.15 | 0 | 0.00 | 0.0045 | 0.07 |
| 2010 | 5.70 | 3.451 | 60.55 | 0.685 | 12.02 | 0.622 | 10.92 | 0.361 | 6.33 | 0.162 | 2.84 | 0.0209 | 0.37 | 0 | 0.00 | 0.0052 | 0.09 |
| 2011 | 5.83 | 3.366 | 57.73 | 0.935 | 16.03 | 0.656 | 11.25 | 0.543 | 9.31 | 0.123 | 2.11 | 0.0236 | 0.40 | 0 | 0.00 | 0.0189 | 0.32 |
| 2012 | 6.02 | 3.216 | 53.42 | 1.061 | 17.62 | 0.768 | 12.76 | 0.467 | 7.75 | 0.091 | 1.51 | 0.0165 | 0.27 | 0 | 0.00 | 0.0083 | 0.14 |
| 2013 | 5.89 | 2.900 | 49.27 | 1.198 | 20.36 | 0.885 | 15.04 | 0.425 | 7.21 | 0.090 | 1.53 | 0.0432 | 0.73 | 0 | 0.00 | 0.0072 | 0.12 |
| 2014 | 5.58 | 2.570 | 46.06 | 1.407 | 25.22 | 0.854 | 15.30 | 0.482 | 8.65 | 0.077 | 1.38 | 0.0093 | 0.17 | 0 | 0.00 | 0.0062 | 0.11 |
| 2015 | 5.52 | 2.302 | 41.71 | 1.408 | 25.50 | 1.076 | 19.49 | 0.444 | 8.05 | 0.075 | 1.36 | 0.0063 | 0.11 | 0 | 0.00 | 0 | 0.00 |
